# Inequality in mammography uptake: results from recruitment phase of first cohort study among Iranian Kurdish population

**DOI:** 10.1186/s41256-022-00277-9

**Published:** 2022-11-10

**Authors:** Mehdi Mirzaei-Alavijeh, Bonnie Jerome-D”Emilia, Farid Najafi, Mehdi Moradinazar, Razieh Pirouzeh, Farzad Jalilian

**Affiliations:** 1 Social Development and Health Promotion Research Center, Health Institute, Kermanshah University of Medical Sciences, Kermanshah, Iran; 2 School of Nursing, Rutgers, The State University of New Jersey, Camden, USA; 3 Research Center for Environmental Determinants of Health, Health Institute, Kermanshah University of Medical Sciences, Kermanshah, Iran

**Keywords:** Breast cancer, Mammography, Inequalities, Kurdish women

## Abstract

**Background:**

Breast cancer is the most common malignant disease in women and is the leading cause of cancer deaths among women. Mammography is the best and the most available diagnostic method for breast cancer early detection. The aim of this study was to investigate the prevalence and inequality in the mammography uptake among Kurdish women in the west of Iran.

**Methods:**

This cohort study was conducted using data extracted from the Ravansar Non-communicable Cohort Study among Kurdish women in the west of Iran from 2014 to 2018. The sample included 5289 women aged 35–65 years. The relative and generalized (absolute) concentration index (RC and GC, respectively) was used to quantify and decompose socioeconomic inequalities in mammography uptake.

**Results:**

Overall concentration index for mammography was 0.2107, indicating that the mammography uptake concentration was greater in women with a higher socioeconomic status (SES). The predictor variables accounted for 44.6% of the inequality in the mammography uptake. Higher SES, living in urban areas, and age group of 51–55 years old increased the chance of having a mammogram. Available evidence supports the inequality of mammography uptake in favor of women with higher SES.

**Conclusions:**

Cost-free screening services for low SES women, and the development of breast cancer prevention campaigns focusing on disadvantaged women could have an important role in mammography uptake and in the reduction of inequalities.

## Background

Breast cancer is the most common malignant disease in women, the leading cause of cancer deaths among women throughout the world, and one of the major public health problems in both developed and developing countries [1]. According to the American Cancer Society, it is the most diagnosed cancer among women in the United States, resulting in more than 40,000 deaths each year, and the lifetime risk of the disease is about 12.32% [2]. In Iranian women, breast cancer is the most common malignancy [3]. Kazeminia et al. carried out a systematic review and meta-analysis and reported the prevalence of breast cancer in Iranian women was 23.6% (95% CI 15.3–34.7%) [4]. As well as, it should be noted that the clinical stage in the diagnosis of breast cancer is one of the highest prognostic factors for survival; however, in developing countries the greater part of breast cancer are diagnosed in regionally spread stages [5].

Breast cancer has a clinically long latent phase (about 8–10 years) and the early detection of disease can save the patient from the need for extensive treatment death [6]. On this subject, evidence indicated that mammography screening program can be reduces the risk of breast cancer mortality [7]. For example, Kalan Farmanfarma et al. in their systematic review and meta-analysis reported that mammography is the efficient method for breast cancer early detection and about 35–50% of breast cancer might be recognized in the early stages by mammography [8]. Nevertheless, the rate of mammography uptake is low among Iranian women [3]. In Iran, mammography screening is not organized; there is only an opportunistic screening of breast cancer which is recommended that women older than 40 should have an annual mammogram and eligible women in primary healthcare settings are referred to perform mammography; of course, accomplishment of the mammography is not free of charge in Iran [9].

There are two related challenges in Iranian women breast cancer control programs. First, socioeconomic status (SES) has improved in Iran and this in turnhas resulted in a reduction in the size of families and an increased age of first childbirth. Secondly the incidence of breast cancer in young women has increased during recent years [10]. SES is one of the important determinants of health-related behaviors [11]. The term "SES" describes a situation in which a person or group in a vertically structured society, with reference to socioeconomic factors (mainly education, employment, and income) is placed [12]. In recent decades, some evidence suggests that women with higher SES are more likely to uptake a breast cancer screening tests than those with lower SES [13–15].

On the other hand, one of the main goals of health policymakers is to facilitate access to health services so that all groups of society can benefit from these services [16]. Thus, our research aims are: (a) to describe mammography uptake in the west of Iran based on data from a first cohort study in Iranian Kurdish population; (b) to explore factors related to inequality in mammography uptake among Iranian Kurdish women.

## Methods

### Research design and data source

This cohort study was conducted using the data of Ravansar Non-communicable Cohort Study (RaNCD). The RaNCD study is the part of Prospective Epidemiological Research Studies in Iran (PERSIAN), which was coordinated by the Deputy of Research and Technology of the Ministry of Health in Iran. This study was carried out in Kermanshah province on permanent residents from 35 to 65 years of age. Ravansar County is located in western Iran and close to Iraq; the people living in this city are mostly Kurd. The population of Ravansar County is about 50,000. In Ravansar County, there are 3 urban and 2 rural healthcare centers, as well as 32 active local primary health care units (health houses) in rural areas. In the RaNCD study, nearly 10,000 people aged 35–65 years were randomly selected and enrolled in the study [17].

### Questionnaire

The cohort questionnaire was administered by trained interviewers. The questionnaire for assessmentof socioeconomic inequalities in mammography uptake included two sections.

Section One: dependent variable: To assess whether or not the participants had a history of mammography uptake, we used one item “Have you ever had mammography for breast cancer screening” with a response of yes or no.

Section two: independent variables: age groups (35–40, 41–45, 46–50, 51–55, 56–60 and 61–65 years old), marital status (single, married, divorced/widowed), educational level (illiterate, 1–5 years, 6–9 years, 10–12 years, 13 years and more), residence (urban and rural), smoking (yes, no), body mass index (BMI) status (≤ 24.9, 25–29.9, 30–34.9 and > 35), contraceptive drug use (yes, no), and pregnancy number (0, 1–3, 4–5, ≥ 6), and daily physical activity measured by metabolic equivalent of task (METs). The METs of each activity was obtained based on participant self-report. Physical activity levels were classified as low (24–36.5 METs-hours per week), moderate (METs-36.6–44.9 h per week) and heavy (METs- ≥ 45 h per week [18]. Additionally, Socioeconomic Status (SES) index as the main variable representing household economic status was calculated by using Principal Components Analysis (PCA) and taking into account the economic and social variables of the participants. The SES information was related to durable goods and social determinants including ownership of a car, refrigerator, television (s), separate freezers, a washing machine, vacuum cleaner, mobiles, bicycles, laptops, etc., as well as housing,number of rooms in the house, heating, air conditioner, domestic and foreign travel per year.These variables were entered into the PCA model. The population studied was classified according to an SES variable with the following levels by quintile: the poorest, poor, middle, wealthy, and richest, and was used as an indicator for SES in the inequality analysis.

### Data analysis

Data were analysed by STATA software version 14.1. Descriptive statistics were used to summarize and organize the data. Univariate and multivariate analysis using logistic regression were used to ascertain the associated factors for mammography uptake. After performing univariable regression, variables with *p* < 0.3 were included in multivariable analysis, and variables with *p* < 0.05 remained in the multivariable model. SES-related inequality in mammography uptake was estimated by the Concentration Index (CI) and the concentration curve [19]. The concentration curve is a two-dimensional graph. In the horizontal axis, the population cumulative percentage of SES is from the poorest to the richest, and in the vertical axis, it is the cumulative percentage of the dependent variable (mammography uptake). The 45-degree line represents full equality in the distribution of the dependent variable (mammography uptake). If the mammography uptake among groups with lower SES is greater, the focus concentration curve would be located below the equality line and the numerical value of the concentration index would be negative.

The concentration index is extracted from the concentration curve and it equals twice the space between the focus curve and the equality line (45-degree). If the index is zero, this means that the variable was distributed equally among socio-economic groups.The following formula was used to measure inequality and estimate the concentration index:

1 $$CI = \frac{{2*cov\left( {y_{i} r_{i} } \right) }}{\mu }$$

where $y_{i}$ is the dependent variable for the person $i$, *μ* its mean and $r_{i}$ the fractional rank by income. Considering that the dependent variable (mammography uptake) in our study was a binary variable, the concentration index may not fit between-1 and + 1; thus, the concentration index was normalized by dividing the estimated value of the concentration index by $1 - \mu$ [20].

2 $$C_{n} = \frac{{C_{I} }}{1 - \mu }$$

To analyse inequality and to determine the contribution of each of the socioeconomic factors in the development of inequality, the syntactic analysis method was used [21]. This method, based on regression analysis, evaluates the relationship between the dependent variable and the demographic, behavioural and economic determinants that effect the dependent variable. In the present study, partial effect was used to estimate the probability of mammography uptake.

3 $$y = \alpha + \mathop \sum \limits_{k} \beta_{k} x_{k} + \varepsilon$$

Independent variables included age, marital status, level of education, residence (urban, rural), and METs, BMI, oral contraceptive use, number of pregnancies, and socioeconomic status. After estimating the partial effect and estimating the coefficients, the concentration index for y is shown below.

4 $${ }Cn = \mathop \sum \limits_{k} \left( {\frac{{\beta_{k} \overline{x}_{k} }}{\mu }} \right)C_{k} + GC_{\varepsilon } /\mu$$

where C_n_ is the concentration index, µ is the mean of the dependent variable, $\overline{x}_{k}$ is the mean of each of the independent variables, $C_{k}$ is the concentration index for the variable, and GCε is the generalized concentration index for ε.

Based on Eq. 4, the concentration index for the dependent variable is a combination of two components. The first component or $\sum\nolimits_{k} {\left( {\frac{{\beta_{k} \overline{x}_{k} }}{\mu }} \right)C_{k} }$, indicated how the concentration index was explained by the systematic changes of the independent variables in the distribution of socioeconomic groups. A negative contribution of a dependent variable to C_n_ indicated the distribution of wealth-dependent variables and the relationship of this variable to mammography uptake are likely to contribute to less mammography uptake among the poor. The second component or $GC_{\varepsilon } /\mu$ indicated the inequality that is not explained by the systematic changes of independent variables in socioeconomic groups. In our study, normalized CI was decomposed with the following formula.

5 $$C_{n} = \frac{{\mathop \sum \limits_{K} \left( {\frac{{\beta_{K} \overline{X}_{K} }}{\mu }} \right)C_{K} }}{1 - \mu } + \frac{{\frac{{GC_{\varepsilon } }}{\mu }}}{1 - \mu }$$

### Ethical considerations

The research ethics committee of Kermanshah University of Medical Sciences approved the study protocol (KUMS.REC.1394.318). Details of the study were provided to participants, including how the study was being performed, the confidentiality of information, as well as the purpose of study, prior to participation.

## Results

### Demographic characteristics

The mean age of women in the sample was 48.36 years [95% CI 48.13, 48.58], with a range from 35 to 65 years. The majority of participants were illiterate (62.7%) and married (83.8%). About 5.2% of the respondents had history of cigarette smoking. More details of demographic characteristics of the participants are shown in Table 1.

**Table 1:** Demographic characteristics of the participants

Variables	Total N (%)	Mammography N (%)
*Age (year)*		
35–40	1168 (22.1)	67 (5.7)
41–45	1144 (21.6)	147 (12.9)
46–50	927 (17.5)	146 (15.8)
51–55	760 (14.4)	139 (18.3)
56–60	738 (14.0)	90 (12.2)
61–65	552 (10.4)	45 (8.2)
*Marital status*		
Married	4437 (83.8)	591 (13.3)
Single	329 (6.2)	5 (1.5)
Divorced/widow	526 (10.0)	38 (7.2)
*Level of education*		
Illiterate	3318 (62.7)	394 (11.9)
1–5 years	1292 (24.3)	167 (12.9)
6–9 years	316 (6.0)	44 (13.9)
10–12 years	218 (4.1)	18 (8.3)
≥ 13 years	145 (2.7)	11 (7.6)
*Residence*		
Urban	3029 (57.3)	458 (15.1)
Rural	2260 (42.7)	176 (7.8)
*Smoking status*		
No	4999 (94.8)	598 (11.9)
Yes	276 (5.2)	34 (12.3)
*BMI*		
≤ 24.9	1194 (22.6)	99 (8.3)
25–29.9	2141 (40.5)	258 (12.1)
30–34.9	1446 (27.3)	206 (14.3)
> 35	475 (9.1)	69 (14.5)
*Metabolic equivalent of task (METs)*		
24–36.5	1188 (22.5)	152 (12.8)
36.6–44.9	3671 (69.5)	435 (12.2)
≥ 45	528 (10.0)	47 (8.9)
*Oral contraceptive use*		
No	1221 (23.2)	112 (9.2)
Yes	4060 (76.8)	522 (12.9)
*Number of pregnancies*		
0	233 (5.4)	13 (5.6)
1–3	1800 (35.3)	205 (11.4)
4–5	1376 (27.1)	215 (15.6)
≥ 6	1648 (32.2)	197 (11.9)
*Socio-economic status*		
1st quintile (the poorest)	1058 (20.0)	59 (5.6)
2nd quintile	1058 (20.0)	114 (10.8)
3rd quintile	1058 (20.0)	134 (12.7)
4th quintile	1058 (20.0)	134 (12.7)
5th quintile (the richest)	1057 (20.0)	193 (18.3)

According to the findings, 11.99% of the respondents had undergone mammography at least once.

### Socioeconomic inequality in mammography uptake

**Fig. 1:**
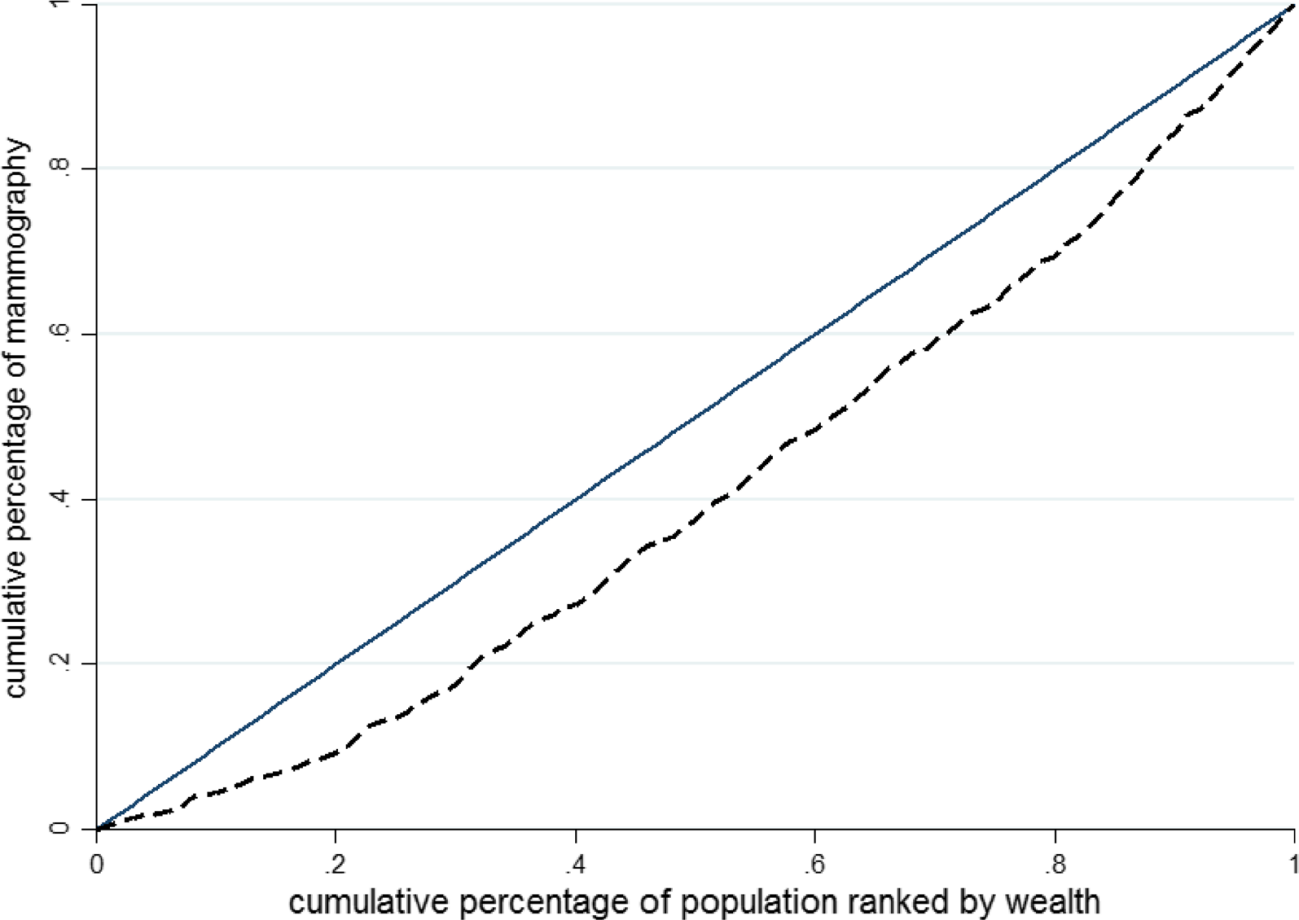
The concentration curves for mammography in women

The overall concentration index for mammography uptake is shown in Fig. 1. As seen in Fig. 1, the overall concentration index for mammography uptake was 0.2107, which indicated that the mammography uptake concentration was greater in women with a higher SES.

### Associated factors of inequality in mammography uptake

The associated factors for mammography uptake is shown in Table 2. Initially, univariate analysis was performed using logistic regression and non-significant variables (marital status, smoking, BMI, METs, oral contraceptive use and number of pregnancies) were removed from the model. The findings of multivariate analysis are also presented in Table 2. As can see in Table 2, the SES, location of residence, level of education and age had significant effects on mammography uptake inequality among the Iranian Kurdish women. Living in urban has increased the chances of uptake a mammogram among women. Also, married women were more likely to have had a mammogram, but it was not statistically significant. Furthermore, our findings show that with increasing education level, the chances of uptake mammogram in women have decreased. Multivariate analysis indicated that among women in 5th quintile (the richest), the chance of mammography uptake was 3.52 (2.49–4.98) times more likely compare with among the 1st quintile women.

**Table 2:** Associated factors for mammography uptake

Variables	Crude OR (95% CI)	P	Adjusted OR (95% CI)	P
*Age (year) ref (35–40)*				
41–45	1.97 (− 2.69–1.68)	< 0.001	2.34 (− 1.71–3.20)	< 0.001
46–50	2.70 (2.03–3.60)	< 0.001	2.85 (2.06–3.95)	< 0.001
51–55	3.47 (2.58–4.67)	< 0.001	3.67 (2.62–5.15)	< 0.001
56–60	2.93 (2.11–4.08)	< 0.001	2.32 (1.61–3.35)	< 0.001
61–65	2.47 (1.60–3.82)	0.001	1.70 (1.09–2.64)	0.001
*Marital status ref (Single)*				
Married	1.28 (0.43–3.79)	0.76	–	–
Divorced/ widow	1.02 (0.33–3.19)	0.83	–	–
*Level of education ref (illiterate)*				
1–5 years	0.93 (0.76–1.14)	0.634	1.00 (0.80–1.26)	0.634
6–9 years	0.90 (0.67–1.22)	0.962	1.06 (0.72–1.55)	0.962
10–12 years	0.48 (0.30–0.76)	0.006	0.62 (0.36–1.08)	0.006
≥ 13 years	0.56 (0.30–1.03)	0.042	0.49 (0.25–0.98)	0.042
*Residence ref (Rural)*				
Urban	1.32 (1.09–1.59)	0.004	1.15 (0.93–1.41)	0.185
*Smoking ref (no)*				
Yes	1.67 (1.13–2.46)	0.053	–	–
*BMI ref (n* ≤ *24.9*				
25–29.9	1.26 (0.98–1.63)	0.056	–	–
30–34.9	1.35 (1.05–1.75)	0.284		
> 35	1.34 (0.92–1.88)	0.313		
*Metabolic equivalent of task (METs) ref (24–36.5)*				
36.6–44.9	0.80 (0.65–0.97)	0.269	–	–
≥ 45	0.64 (0.45–0.90)	0.129		
*Oral contraceptive use ref (no)*				
Yes	0.78 (0.61–0.98)	0.056	–	
*Number of pregnancies ref (no pregnant)*				
1–3	1.06 (0.56–2.00)	0.879	–	–
4–5	1.71 (0.91–3.22)	0.672		
≥ 6	1.84 (0.97–3.48)	0.793		
*Socio-economic status ref (1st quintile)*				
2nd quintile	1.27 (0.96–1.68)	0.149	2.03 (1.45–2.84)	0.149
3rd quintile	1.38 (1.04–1.83)	0.028	2.29 (1.64–3.19)	0.028
4th quintile	2.00 (1.52–2.64)	< 0.001	2.25 (1.60–3.16)	< 0.001
5th quintile (the richest)	1.67 (1.21–2.29)	< 0.001	3.52 (2.49–4.98)	< 0.001

The decomposition of socioeconomic-related inequality towards mammography uptake is shown in Table 3. SES status was the strongest determinant of mammography uptake inequality, explaining approximately 42% of the observed inequality. From the total contribution of SES, women in the 5th quantile (richest) had a contribution equal to 40.24%.

**Table 3:** Decomposition of socioeconomic-related inequality towards mammography uptake

Variables	Elasticity	C_k_	Absolute	Percentage	Sum percentage contribution
*Age (year) ref (35–40)*					
41–45	1.4703	0.0753	0.1259	2.6521	− 3.8769
46–50	1.4662	0.0371	0.0618	1.3011	
51–55	1.5047	− 0.0673	− 0.1150	− 2.4227	
56–60	0.9169	− 0.1312	− 0.1367	− 2.8804	
61–65	0.4120	− 0.2562	− 0.1199	− 2.5270	
*Marital status ref (Single)*					
Married	0.5083	0.1887	0.1090	2.2968	2.3390
Divorced/ widow	1.0873	0.0016	0.0020	0.0422	
*Level of education ref (illiterate)*					
1–5 years	0.0119	0.1979	0.0027	0.0565	− 5.2122
6–9 years	0.0368	0.4349	0.0182	0.3829	
10–12 years	− 0.1763	0.6249	− 0.1252	− 2.6375	
≥ 13 years	− 0.1531	0.8221	− 0.1430	− 3.0140	
*Residence ref (Rural)*					
Urban	1.7327	0.2016	− 0.3969	8.3636	8.3636
*Smoking ref (no)*					
Yes	0.1066	− 0.2739	− 0.0332	− 0.6992	− 0.6992
*BMI ref (n* ≤ *24.9*					
25–29.9	0.5175	0.0266	0.0157	0.3299	1.0319
30–34.9	0.3382	0.0831	0.0319	0.6729	
> 35	0.1709	0.0071	0.0014	0.0291	
*Metabolic equivalent of task (METs) ref (24–36.5)*					
36.6–44.9	− 0.5287	0.0226	− 0.0135	− 0.2855	0.9172
≥ 45	− 0.1677	− 0.2995	0.0571	1.2027	
*Oral contraceptive use ref (no)*					
Yes	− 0.6861	0.0216	− 0.0168	− 0.3542	− 0.3542
*Number of pregnancies ref (no pregnant)*					
1–3	0.3246	0.1346	0.0497	1.0463	0.1786
4–5	0.3624	0.0469	0.0193	0.4072	
≥ 6	0.3041	− 0.1751	− 0.0605	− 1.2750	
*Socio-economic status ref (1st quintile)*					
2nd quintile	1.1861	− 0.4000	− 0.5390	− 11.3574	41.9086
3rd quintile	1.3852	0.0002	0.0003	0.0063	
4th quintile	1.3579	0.4003	0.6177	13.0149	
5th quintile (the richest)	2.1005	0.8003	1.9101	40.2448	
	0.1199	0.2107			44.5963

## Discussion

The objective of the current study was to determine whether SES inequality was related to mammographyuse among Kurdish women in the west of Iran. According to the findings, 11.99% of Kurdish women had undergone mammography at least once. Sakkaki et al. (2014) stated in their study that 13.1% of women who were referred to health centres in Zanjan County (in the north-west of Iran) had undergone mammography [22]. Also, Rejali et al. (2018), in a study of 9591 women aged 20–65 years old in Isfahan, in central Iran reported that 15.7% of the subjects had mammography at least once [23]. Furthermore, Mirzaei-Alavijeh et al. (2018) in their study of women in the west of Iran indicated that 13% of the women had at least one mammogram [3]. Al-Wassia et al. (2017) reported that 40% of Saudi women aged ≥ 40 years had at least one mammogram [24]. Moreover, Elias et al. (2017), in a study of 2400 Lebanese women reported that 45% of them had at least one mammogram [25]. It should be noted that women's cancer screening uptake campaigns in Asia are necessary because mammography uptake rates are lower compared to developed countries: for example rates are 75% and 83% among women in Australia and Scotland, respectively [26]. Furthermore, a comparison of our findings with similar research outside of Iran [24–26] indicated the mammography rates, among Iranian Kurdish women, similar to other ethnicities in Iran, are much lower when compared to other countries. These findings could be a warning to health policymakers in Iran thathealth promotion activities should focus on a widespread screening program for breast cancer, which is the most common cancer in women in Iran.

Our results indicated that the overall concentration index for mammography was 0.2107; which demonstrates that the mammography uptake concentration is greater in women with a higher SES. In addition, the predictor variables accounted for 44.6% of the observed inequality in the mammography uptake. Moreover, our findings indicated the SES, residence, level of education, and age had significant effects on inequality in mammography uptake among the Kurdish women. In fact, SES was the strongest determinant of mammography uptake inequality (42%) with major contribution of 5th quintile (40.24%). The results of similar studies are in line with our finding. For example, Elias et al. (2017) in their study indicated that higher SES was significantly associated with ever having had mammography screening^26^. In addition; our findings are in line with studies from France [27], Mexico [28], Great Britain [29], and Italy [30]. In line with our findings Calo et al. in their study in Houston, Texas indicated that individuals who live in more socioeconomically disadvantaged areas are less likely to uptake cancer screening services [31]. It should be noted, organized mammography screening not recommended in low-income countries due to high costs, and a more cost-effective way is breast cancer early diagnosis [5]. Cost-free screening services for low SES women, and the development of breast cancer prevention campaigns focusing on disadvantaged women (increasing women's awareness of the symptoms of cancer) could have an important role in mammography uptake and in the reduction of inequalities.

Location of residence, with a contribution of 8.36% to total observed inequality, had the second highest impact. Our finding is in line with studies from other Middle Eastern countries. For example, differences in residence and mammography uptake in the study of Al-Wassia et al. (2017) among Saudi women have been also reported [24]. Leung et al. (2015) carried out a study on women in Scotland and Australian and reported that rural women were not less likely to have received a mammogram when uptake compared with urban women [26]. In addition, Maheswaran et al. (2006) in their studies among 34,868 women aged 50–64 years in North Derbyshire, UK, reported no difference between urban and rural areas in uptake of breast cancer screening [32]. However, in the US, studies have found that women residing in rural areas have lower rate of mammography [33, 34]. It seems the provision of services to rural women in developed and developing countries have had mixed results in terms of mammography uptake.

A notable result in the present study was that with an increase in the level of education, the likelihood of mammography declined. Whereas other studies have found that increasing the level of education is expected to increase the probability of cancer screening behaviors [35, 36]. It is relevant to note, women with higher education in our study were younger. In this regards, Katapodi et al. in their meta-analytic review reported perceived risk of breast cancer is influenced by age [36]. As well as, evidence indicated that people rarely adopt precautions when they do not believe they are at risk [37]. This evidence suggests that raising awareness of breast cancer risk is essential, especially among younger women and could be useful in order to increase screening behaviors such as mammography uptake.

This study has some limitations. First, our information about mammography among women were based on self-report and therefore may be from recall bias. Second, this study was conducted among Kurdish women in the west of Iran and therefore generalizability of our findings is limited, we suggest investigating inequalities between all ethnicities in Iran. Third, some of the observed significant statistical effects can be due to the large sample size of our study, thus, future studies are necessary to confirm our findings.

## Conclusions

Identifying the determinants of breast cancer screening behaviors such as mammography is an important public health issue. The present study indicated that the higher SES and residence in urban areas positively contribute to the observed inequality in mammography uptake among Kurdish women in the west of Iran. Develop organized screening programs, a wider spread of cost-free screening services, and the development of breast cancer prevention campaigns by focusing on disadvantaged women could play an important role in mammography uptake and the reduction of inequalities in Iran.

## Data Availability

The datasets generated during the current study are available from the corresponding author on reasonable request.

## Abbreviations

BMI: Body mass index
CI: Concentration index
RaNCD: Ravansar non-communicable cohort study
SES: Socioeconomic status
METs: Metabolic equivalent of task
